# Can Driving-Simulator Training Enhance Visual Attention, Cognition, and Physical Functioning in Older Adults?

**DOI:** 10.1155/2018/7547631

**Published:** 2018-02-07

**Authors:** Mathias Haeger, Otmar Bock, Daniel Memmert, Stefanie Hüttermann

**Affiliations:** ^1^Institute of Physiology and Anatomy, German Sport University Cologne, Am Sportpark Müngersdorf 6, 50933 Cologne, Germany; ^2^Institute of Training and Computer Science in Sport, German Sport University Cologne, Am Sportpark Müngersdorf 6, 50933 Cologne, Germany

## Abstract

Virtual reality offers a good possibility for the implementation of real-life tasks in a laboratory-based training or testing scenario. Thus, a computerized training in a driving simulator offers an ecological valid training approach. Visual attention had an influence on driving performance, so we used the reverse approach to test the influence of a driving training on visual attention and executive functions. Thirty-seven healthy older participants (mean age: 71.46 ± 4.09; gender: 17 men and 20 women) took part in our controlled experimental study. We examined transfer effects from a four-week driving training (three times per week) on visual attention, executive function, and motor skill. Effects were analyzed using an analysis of variance with repeated measurements. Therefore, main factors were group and time to show training-related benefits of our intervention. Results revealed improvements for the intervention group in divided visual attention; however, there were benefits neither in the other cognitive domains nor in the additional motor task. Thus, there are no broad training-induced transfer effects from such an ecologically valid training regime. This lack of findings could be addressed to insufficient training intensities or a participant-induced bias following the cancelled randomization process.

## 1. Introduction

Many studies strongly suggest that attention declines with advancing age. This has been documented via several components of visual attention, such as selective attention [1, 2], sustained attention [1], distributed attention [3, 4], and divided attention [2]. There is also a decline of executive functions in old age, which is indicated by reduced inhibitory control, declined working memory, or slower cognitive flexibility [5–7]. The age-related deficits of executive functions [7–10] and visual attention can be ameliorated by practice [11, 12]. Thus, the purpose of our study is to evaluate a training approach for these cognitive functions.

Cognitive functions can be trained by classic cognitive training [13], computerized brain training programs [12], and video games [7, 14–18] (for a corresponding review, see [19]). A further review in healthy older adults showed that computerized training could improve working memory, cognitive speed of processing, or visuospatial abilities, but not executive functions or attention [20]. The authors conducted domain-specific analyses for cognitive functions but neglected differences regarding the kind of computerized training. Thus, different types of programs might have different effects since action video games, for example, are probably effective based on fast reactions and management of multiple tasks at the same time [21].

It has been suggested that cognitive training based on video/computer games is more attractive than cognitive training based on standard laboratory tasks, as it is diverse and motivating rather than stereotyped and dull [22–24]. However, training benefits seem to be smaller rather than larger if compared to laboratory-type training [19, 22, 25]. One possible explanation is that trained activities were not familiar and realistic; that is, they lacked ecological validity [26, 27]: the cognitive benefits of training regimes are thought to increase when trained activities are similar to situations of everyday life [28]. Thus, an ecologically valid training reflects everyday actions and could be helpful, especially for older adults, to pass demanding situations (i.e., driving on dangerous crossroads). In such situations, people also have to manage multiple tasks at the same time, and studies including multitasking training have already showed cognitive benefits [14].

One possible approach for a diverse, motivating, and ecologically valid cognitive training regime is driving in a car simulator. It requires selective, sustained, distributed, and divided attention as well as executive abilities, that is, cognitive skills which are known to decay in older age (see above). Furthermore, there is some evidence that visual attention [29, 30] and working memory [31] are connected with real-life driving performance. Although two earlier studies found little benefits of driving-simulator training for older participants' attention, training quantity was limited to 10 × 40 minutes in one [23] and 2 × 120 minutes in the other study [32]. Additionally, results from Casutt and colleagues [23] only showed effects in an overall cognitive score with the greatest impact on simple and complex choice reaction times. It remains questionable whether components of visual attention were improved. Furthermore, task realism and benefits for visual attention were reduced by using only one central screen to display the driving scenarios. We believe that more substantial benefits of driving-simulator training are conceivable since they already have been observed in reciprocal approaches, in which cognitive training targeting speed of processing [11, 32, 33] or attention and dual-tasking training [34] improved driving performance. In our approach, we want to reevaluate the cognitive benefits of driving-simulator training using more and longer sessions as well as a larger visual display. We reasoned that a larger display may provide a more immersive experience and thus facilitate top-down modulation of visual attention (i.e., upcoming moving obstacles near the lane), which was recently connected with the prefrontal cortex (PFC) [2, 35]. Furthermore, there is growing evidence that the PFC moreover regulates the focus of attention, selects information, and controls executive functions [36]. So, we decided to expand the scope of outcome variables and include executive functions. Besides the theoretical connection, executive functions are known to be associated with driving performance [37] but have not been evaluated yet in the context of driving-simulator training.

A further aspect that has also not been evaluated before is functional mobility as a far transfer from driving-simulation training. We took that into consideration because driving involves limb movements and requires complex visual processing—two abilities associated with mobility and risk of falls in older adults [38–40]—and also because there is an association between cognition (i.e., executive function and dual tasking) and gait parameters (i.e., walking speed) [41]. So, we decided to assess functional mobility by the Timed Up-and-Go (TUG) test, an established marker of reduced mobility [42]. From our point of view, it is an interesting insight concerning a relationship between a computerized training (i.e., including cognitive and coordinative aspects) and a functional parameter, which was slightly examined before [43].

Summing up, we reevaluated driving-simulation training in older adults [23, 32] to extend existing knowledge about effects on visual attention, executive functions, and mobility. Since other computerized training regimes showed benefits on different cognitive functions [19, 20, 44], we hypothesized that our ecologically valid training (1) would improve different parameters of visual attention (distributed, divided, selective, and sustained attention); (2) would improve core executive functions (working memory and task switching); and (3) would show a positive far transfer from cognitive training on functional abilities, as it is suggested in literature [43].

## 2. Method

### 2.1. Sample

Required sample size was calculated by G-Power® 3.1 as follows: earlier attention studies yielded effect sizes ranging from *f* = 0.105 to *f* = 0.47 [14–16, 23, 32], so we expect an effect size of *f* = 0.25. For the interaction term of a 2 (group) × 2 (time) analysis of variance with *f* = 0.25, alpha = 0.05, and beta = 0.80, correlation among repetitive measures = 0.40, G-Power yielded a required total sample size of 40 participants. Participants were recruited by distributing flyers on the university campus and, in the city, sending them out through mailing lists, uploading them on the Internet, and adding them to newspapers.

**Figure 1:**
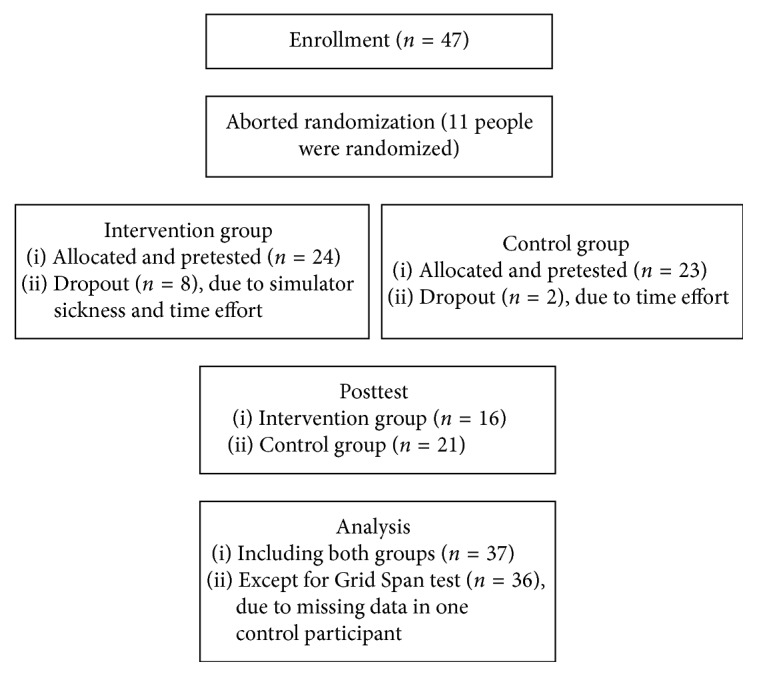
Study flowchart describing participants' allocation in both groups. Enrollment started in November 2015, and the study was finished in September 2016.

We initially planned a randomized controlled trial, using a computerized block-randomized (two per block, balanced for groups) allocation; however, we had to abort this process after a few participants because a lot of interested people refused their participation (i.e., they only were interested in one: intervention or control group). Additionally, recruitment was slow and the dropout rate was high because of the high time demand of this study. So, we asked all respondents personally who met our inclusion criteria to take part in the intervention group, and if they refused because of the high time demand, we asked them to participate in the control group. There was no additional investigator blinding. Finally, we had a controlled experimental design without randomization (Figure 1).

Inclusion criteria were 65 to 80 years of age, no neurological disease, MMSE >23, normal or corrected-to-normal vision, and car driving experience of no more than 20 hours per month within the last six months. Exclusion criteria were a low MMSE score (<24), previous or actual neurological diseases (i.e., stroke, multiple sclerosis), and daily driving routine. These criteria were formulated to bring in older, not cognitive-impaired drivers with minor driving routine. It was met by 47 respondents, of whom four dropped out later on because of time limitations and six because of simulator sickness. In effect, 16 men and women were retained in the intervention and 21 in the control group.

### 2.2. Training

**Figure 2:**
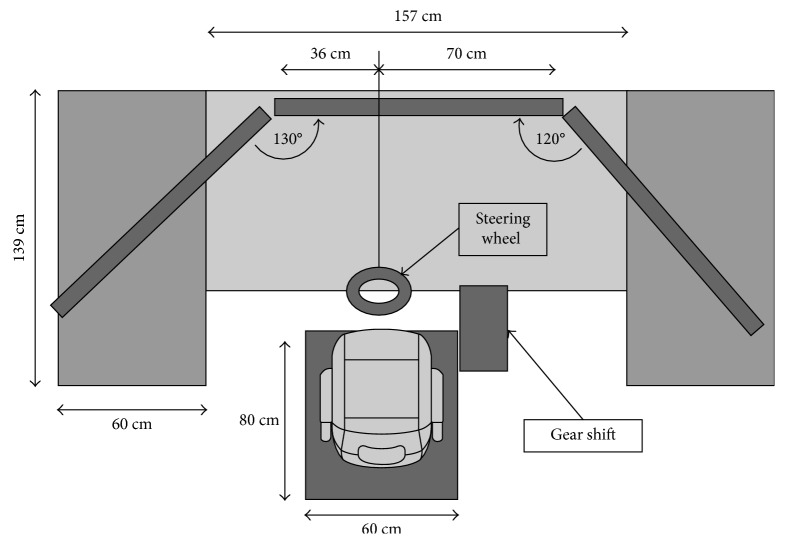
Top view of the driving simulator including tables, monitors, seat, gear shift, and steering wheel. Pedals are under the table (not shown).

We used a commercially available driving simulator (Carnetsoft BV, Groningen, NL) which consists of a computer, three rendering monitors, a steering wheel, pedals, and a gear shift (Figure 2). The monitors have a 48″ diagonal and a 100 HZ frame rate and were positioned on laboratory tables at eye level in front of a black shroud which blocked vision of the laboratory room. Pedals and the driver's seat were adjusted individually for comfort. Steering wheel, seat, pedals, and gear shift were placed mid between the center and the left edge of the middle screen to imitate the drivers' position in a real car.

The Carnetsoft® software includes a curriculum with multiple driving scenarios from which the following were used for training: learn to drive, using a gear shift, emergency breaking in different road settings, driving ecologically, noticing road signs during the drive, and danger of driving after consuming alcohol. Scenarios consist of rural areas, towns, highways, or combinations of them and include leading or oncoming vehicles. During the drive, a female voice gave driving directions. In addition, participants received short informal feedback from the instructor after each session (i.e., driving errors). Therefore, we recorded some driving parameters (i.e., velocity and lane-adherence) as well as errors (i.e., missing a stop sign) and reported those afterwards. Additionally, we yielded information for safer driving (i.e., scanning the upcoming lane for potential danger). Training took four weeks with three training sessions of about 50–60 minutes per week. It began with simple scenarios whose difficulty level increased gradually in three main steps. So, the first session was for familiarization of our participants with the car dynamics of the driving simulator (i.e., using pedals and gear shift). The following five sessions had more complex requirements: participants had to enter motorways, overtake slow driving cars, or drive over a longer period. During those scenarios we increased traffic (i.e., approaching cars and slower cars ahead), driving durations, and complexity of scenarios (i.e., from a rural area to a city including pedestrians). Finally, the last six sessions consisted of a randomized order of more challenging tasks: participants should drive while paying attention to traffic signs (i.e., we presented additional signs on the left and right display), long highway driving sessions including a lot of traffic as well as traffic jams, and brake-reaction tasks. During these scenarios, we additionally recorded reaction times (i.e., brake reaction or seeing traffic signs) to inform participants about their results in each session (see above: feedback). A single training session took place during the week between 9 a.m. and 4 p.m., depending on availability of our participants.

### 2.3. Outcome Measures

The following test battery was administered before and after training in the intervention group and four weeks apart in the control group:

- The Precue task [45, 46] is a measure of distributed/spatial attention. Participants respond after a correct, false, or neutral cue to visual stimuli presented on the right or left side of a central fixation point. Performance is quantified as a mean reaction time for correct, false, and neutral cues.
- The D2-Attention task [47] is a measure of selective and sustained attention. In a computerized version of this test, participants watch a sequence of items from the list {d” d' d d' d” d” p” p' p p' p” p”} and have to select all instances of a letter “d” followed by two dashes (d” d” d”) over a time period of six minutes. Performance is quantified as the number of correct answers minus the number of errors.
- The Attention Window task [4, 48] is a measure of multistream divided attention [2]. Participants watch a sequence of two simultaneously presented patterns, each consisting of four objects, dark or light grey triangles and circles. Following the presentation of each pair, they are asked to indicate the number of light grey triangles in both patterns, without being pressed for time. Successive pattern pairs vary quasi-randomly in the number of light grey triangles and in the distance from a central fixation point. Performance is quantified as percentage of correct responses to both patterns in a pair on each axis (diagonal, horizontal, and vertical).
- The Grid Span task [49–51] is a measure of spatial working memory which is related to executive control. Participants watch a sequence of crosses in a 4 × 4 grid and are asked to replicate the sequence immediately thereafter. Sequence length increases from trial to trial, and performance is quantified as length of last correctly replicated sequence.
- The Switching task [52] is a measure of executive flexibility. Participants watch a sequence of small and large fruits and vegetables and have to indicate either the size (task A) or their nature (fruit versus vegetable; task B). In single blocks, only one task is asked for; in switching blocks, the task sequence AABBAABB and so on is asked for. Performance is quantified as mean reaction time in each task.
- The PAQ-50+ [53] is a retrospective physical activity assessment, covering the preceding four weeks.
- The Timed Up-and-Go task [42, 54] is a functional test of gait and balance. Participants stand up from a chair upon command, walk three meters, turn, and walk back to sit down again, all with their habitual velocity. Performance is quantified as mean completion time across three test repetitions.
- The Mini-Mental State Examination (MMSE) [55] was administered as a screening tool on pretests only.

### 2.4. Procedure

The study was preapproved by the Ethics Committee of the German Sport University Cologne, and all participants signed an informed consent before testing started. Each participant was tested individually. During an initial interview, participants were informed about the test battery. Control persons were told that a follow-up evaluation will check whether performing the test battery had lasting effects, and training persons were told about the driving intervention and its possible effects. In sum, testing took approximately one and a half hours. Each session took place during the week between 9 a.m. and 5 p.m., and we tried to maintain the individual testing times from pre- to posttesting.

First, MMSE and PAQ-50+ were completed in the form of an interview. Next, computerized versions of Precue, D2-Attention, Grid Span, Switching, and Attention Window tasks were administered in a randomized order, which was the same during pre- and posttest. Finally, the TUG was conducted three times using a manually operated clock and a chair without armrest.

We preregistered our study in the Open Science Framework (OSF) but unfortunately had to change two methodological aspects later on. Due to slow participant recruitment and limited availability of laboratory space, we had to give up randomized group assignment and cancel the retention test twelve weeks after training.

### 2.5. Statistics

Data from the D2-Attention task, each axis of the Attention Window task, the Grid Span task, and TUG were submitted to a 2 (group: intervention and control) × 2 (time: pre and post) analysis of variance (ANOVA) with repeated measures on the latter factor. For the Switching task, we used a 2 (group) × 2 (time) × 3 (trial: single, nonswitching, and switching) ANOVA with repeated measures on the latter two factors. For the Precue task, we conducted a 2 (group) × 2 (time) × 3 (cue: correct, neutral, and false) ANOVA with repeated measures on the latter two factors. Furthermore, we conducted the Mauchly test for sphericity for the Switching task and the Precue task and used a Greenhouse–Geisser correction in case of a sphericity violation. Regarding this number of tests, we conducted a Bonferroni–Holm correction for multiple testing to adjust *p* values. Training benefits should emerge as significant group × time interactions in these analyses. Interaction effects (i.e., group × time) represent our primary outcome; main effects of these tests are secondary outcome.

As further secondary outcome, we analyzed participants' characteristics using an independent *t*-test, a two-dimensional (group and education) chi-square test, and a 2 (group) × 2 (time) ANOVA to reveal differences between our groups.

## 3. Results

Table 1 shows the demographic characteristics of all participants (*n* = 37) included in data analysis. None of the scores differed significantly between groups at the pretest. Furthermore, there were no group- or time-dependent effects in subjective physical activity (*F*_(1,35)_ = 0.96, MSE = 1516.202, *p* > 0.05).

**Figure 3:**
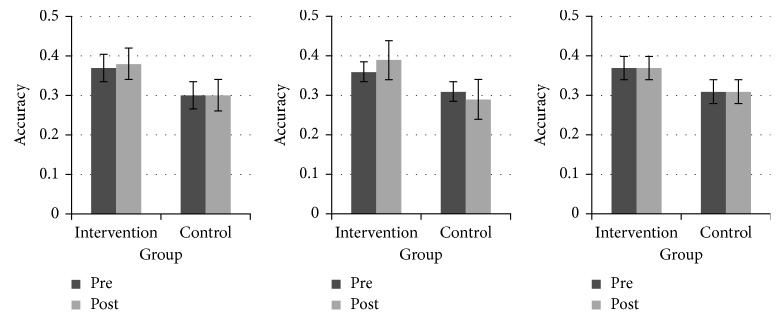
Response accuracy in the Attention Window test, plotted separately for the three axes and for the intervention group and the control group. Boxes indicate across-participant means and error bars the pertinent standard errors. (a) Diagonal. (b) Horizontal. (c) Vertical.

As expected, the Precue task yielded with Greenhouse–Geisser correction (*χ* ^2^_(2)_ = 7.01, *p*=0.03, *ε* = 0.843) a significant main effect for cue (*F*_(1.69,59.01)_ = 19.75, MSE = 786.476, *p* < 0.01). There were no other significant differences, notably not for group × time × cue (*F*_(1.70,59.62)_ = 0.79, MSE = 523.931, *p* > 0.05). The D2-Attention task yielded a significant main effect for time (*F*_(1,35)_ = 13.25, MSE = 418.892, *p* < 0.01), but no other significant effects, in particular not for group × time (*F*_(1,35)_ = 0.00, MSE = 418.892, *p* > 0.05). For the Attention Window task, we found a significant group × time interaction for the horizontal axis (*F*_(1,35)_ = 4.46, MSE = 0.003, *p*=0.04, *η* ^2^ = 0.113); however, this effect did not remain after Bonferroni–Holm correction. There was also neither a significant effect for the other two axes (diagonal: *F*_(1,35)_ = 0.14, MSE = 0.008, *p* > 0.05, *η* ^2^ = 0.004; vertical: *F*_(1,35)_ = 0.01, MSE = 0.007, *p* > 0.05, *η* ^2^ = 0.000) nor any other effects.  Figure 3 illustrates that, in the Attention Window task, accuracy on the horizontal axis increased from pre to post in the intervention group but decreased in the control group; this tendency was absent on the other two axes.

There were no significant effects on the Grid Span task, notably no significant group × time interaction (*F*_(1,34)_ = 1.86, MSE = 0.627, *p* > 0.05). The Switching task yielded with Greenhouse–Geisser correction (*χ* ^2^_(2)_ = 17.03, *p* < 0.01, *ε* = 0.717) significant effects for trial (*F*_(1.44,50.22)_ = 37.39, MSE = 12091.056, *p* < 0.01) and trial × time (*F*_(1.61,56.33)_ = 3.71, MSE = 5061.153, *p*=0.04); but again after Bonferroni–Holm correction the trial × time interaction did not remain significant (*p* > 0.01). Finally, the Timed Up-and-Go task yielded no significance, notably not for group × time (TUG: *F*_(1,35)_ = 3.72, MSE = 0.291, *p* > 0.05).  Table 2 summarizes all outcome scores. Further statistical outcomes are presented in an additional table as Supplementary Material (Table S1).

## 4. Discussion

We evaluated a four-week training program in a driving simulator with a wide field of view, administering three sessions of about one hour duration per week. Outcome measures comprised visual attention, executive functions, and physical abilities. Because of recruitment problems and a 28% dropout rate in the intervention group due to simulator sickness, only 16 participants completed in the intervention group and 21 completed in the control group. We found significant benefits of training for divided visual attention along the horizontal axis; however, statistical significance disappeared after correction for multiple testing. Furthermore, we did not find any significant effects neither for other cognitive measures nor for functional mobility. The lack of more substantial training benefits cannot be attributed to group differences regarding demographic, cognitive, or physical baseline scores (Tables 1 and 2).

First, we will discuss our results in relation to other driving-simulator studies. Casutt and colleagues [15] described a significant training benefit on overall cognitive performance, but regarding visual attention, those authors presented only descriptive statistics with small effects (*d* = 0.13–0.31) for selective attention, field of vision, and divided attention. Roenker and colleagues [32] described no training benefits on the Useful Field of View (UFOV®), a test of various aspects of visual attention and perception. The present study is therefore in line with earlier work since training had no substantial benefits for most cognitive functions. In a first analytical step, we found benefits for the horizontal component of divided visual attention, which did not remain after a further statistical correction. However, we calculated an effect size for that component (overall effect size for divided attention, *η* ^2^ = 0.039; conversion according to [56, 57]) as *d* = 0.403, which is slightly more than the value reported by Casutt et al. [23]. We attribute this stronger effect in our study to the dramatically wider field of view of our simulator (see Introduction).

Our results can also be compared to those on video-game training. Action video games, characterized by moving objects, fast responses, and multiple tasks [21, 58], led to improvements in selective [16] and sustained attention [14], but this was not necessarily the case for other types of video games: a review of computerized cognitive training in older adults confirmed training benefits for visuospatial abilities but not for attention [20]. However, the authors of this review did not differentiate, for example, between computerized cognitive training (e.g., [12]) and video games (e.g., [15]). Especially, in the case of visual attention, a more detailed differentiation would be needed since action video games appeared to be more effective than “slower” video games [21]. So, for action video games, a recent review showed moderate benefits for older adults in attention and visuospatial abilities [44]. We assume that, in our study, improvements of attention were limited since fast responses and multitasking occurred less frequently than in action video games.

Regarding our results, we have to reject our first hypothesis that there is a broad impact on older people's visual attention from our driving-simulator training. This is partly in line with previous driving-simulator studies and “slower” video games.

Unlike earlier driving-simulator studies, earlier video game research also evaluated the effects of training on executive functions. Improvements of working memory [14] and task switching [59] were reported, but a generalized effect on executive functioning is still under discussion [19, 20]. However, a recent review described moderate effects on executive functions from action video games [44]. Thus, in executive functions, the same differentiation like in visual attention might be necessary. We found no effects of driving-simulator training in executive functions, and our data are therefore in agreement with the more pessimistic views. In view of these results, we also had to reject our second hypothesis that driving-simulator training would induce transfer effects on executive functions. The use of engaging and ecologically valid training regimes seems not enough to ensure improved executive functions. Possibly, training has to specifically address those functions, as was the case in the studies by Anguera et al. [14] and Montani et al. [59], since the transfer of training benefits to unpracticed tasks may be limited [60].

Another point of criticism pertains to the software used: the simulated driving tasks were possibly not difficult enough to challenge participants' executive and attentional abilities. At last, we had only six sessions including complex situations that might be not enough for experienced drivers. There should be more visual stimulation to facilitate top-down modulation, which forms an important part in visual attention and executive functions [2, 36]. Further studies should also record participants' training sessions to analyze the training progress. Thus, it would be possible to regulate the training process individually.

In view of functional mobility, we observed no training benefits for participants' as assessed by the TUG test. We again conclude that our ecologically valid training regimes are no guarantee for a strong transfer of training benefits to untrained abilities. So, taking into account that there were also no cognitive training benefits, we rejected our third hypothesis of a positive far transfer from cognitive training on functional abilities.

There are also a few more methodological aspects that should be discussed. First, our recruited older participants were healthy, physically active, and still able to drive a car. Regarding this, benefits might only occur in more inactive people [61], and further studies should also control whether participants additionally use their car during the intervention. Secondly, we used an inactive control group and aborted our randomization process. Therefore, possible differences in motivation, expert knowledge (i.e., in computerized training), or individual arrangements of physical activities (i.e., an inactive control group has more leisure time) could affect our measurements. Further studies should take these points into account.

## 5. Limitations

We preregistered our study protocol in the Open Science Framework (OSF) but have to indicate some methodological changes. First, we encountered substantial recruitment problems because of the time and effort involved in participating. As a consequence, we had to cancel the planned randomization and instead assigned the first 24 participants to the intervention group. Second, six participants from the intervention group dropped out because of simulator sickness. Third, only a few participants were willing to undergo follow-up testing, and we therefore had to cancel that part of our study. Possibly, research with older participants became so popular in recent years that the willingness of older persons to contribute to yet another study has been overstrained. As a consequence of these methodological issues, there could also be a bias based on group-related differences: possibly, our intervention group was more familiar with a computerized training, more motivated, or there occurred group differences in other driving-related traits. Regarding the last point, we also missed to analyze personality traits (e.g., motivation, self-efficacy, and driving behavior) which could further explain differences between our groups. We also should have chosen fewer (in view of multiplicity of analyses) and perhaps other cognitive (i.e., a driving-related dual task) or functional tests (i.e., leg/hand coordination task). At last, we did not save results from individual training sessions for a further analysis (i.e., to detect learning curves); in this regard, it would also be beneficial to evaluate motivation during the training process since our tasks were too easy and motivation possibly dropped as time passes.

## 6. Conclusion

We found no evidence that our diverse and realistic driving-simulator training would improve attention, executive functions, and functional mobility. The only marked training benefit was the one on the horizontal component of divided attention, probably because this component was specifically trained in our horizontally wide display; however, it did not remain after statistical corrections. Perhaps, this lack of findings in our study could be based on a range of methodological aspects. For example, a more complex training including fast reactions and an individual training progress might be more stimulating for visual attention and executive functions. So, in view of further diverse and ecologically valid interventions, the training process and other methodological aspects should be reflected.

## Figures and Tables

**Table 1:** Mean values (and standard deviation) of demographic characteristics, MMSE, and PAQ-50+ scores in the intervention and control group.

		Intervention	Control	Statistics
Age (years)		70.25 (±3.77)	72.38 (±4.17)	*t*_(35)_ = −1.61, *p* > 0.05
Gender (men/women)		8/8	9/12	—
Education (1/2)		8/8	9/12	*c* ^2^_(1,*N* = 37)_ = 0.19, *p* > 0.05
Driving time (hours per month)		10.63 (±7.21)	9.88 (±8.39)	*t*_(35)_ = 0.02, *p* > 0.05
MMSE (score)		28.63 (±1.09)	28.62 (±1.20)	*t*_(35)_ = 0.28, *p* > 0.05
PAQ-50+ (MET/week)	Pre	126.37 (±67.60)	125.35 (±66.84)	T: *F*_(1,35)_ = 2.66, *p* > 0.05, *η* ^2^ = 0.071 G^∗^T: *F*_(1,35)_ = 0.96, *p* > 0.05, *η* ^2^= 0.027
	Post	102.50 (±39.06)	119.40 (±65.76)	

Statistics included *t*-tests, chi-square test (for education: 1 = A level, 2 = O level), and ANOVA (G = group effects, T = time effects) to analyze group differences.

**Table 2:** Mean values and standard deviation of pre- and posttest scores in the intervention and control group as well as statistical results (T = time, C = cue, G = group, Tr = trial).

Test		Intervention	Control	Statistics
Precue “false” (ms)	Pre	371.63 (±64.96)	393.75 (±71.48)	T: *F*_(1,35)_ = 0.15, *p* > 0.05, *η* ^2^ = 0.004 T^∗^G: *F*_(1,35)_ = 0.05, *p* > 0.05, *η* ^2^ = 0.001 C: *F*_(1.69,59.01)_ = 19.75, *p* < 0.00, *η* ^2^ = 0.361 C^∗^G: *F*_(1.69,59.01)_ = 1.68, *p* > 0.05, *η* ^2^ = 0.046 T^∗^C: *F*_(1.70,59.62)_ = 0.46, *p* > 0.05, *η* ^2^ = 0.013 G^∗^T^∗^C: *F*_(1.70,59.62)_ = 0.79, *p* > 0.05, *η* ^2^ = 0.022
	Post	374.38 (±76.03)	391.77 (±77.68)	
Precue “neutral” (ms)	Pre	362.88 (±61.16)	377.48 (±60.41)	
	Post	355.16 (±50.90)	372.71 (±66.04)	
Precue “correct” (ms)	Pre	358.92 (±68.31)	357.26 (±41.64)	
	Post	348.70 (±53.34)	359.86 (±63.12)	
D2 (score)	Pre	141.13 (±37.81)	134.29 (±36.76)	T: *F*_(1,35)_ = 13.25, *p* < 0.01, *η* ^2^ = 0.275 G^∗^T: *F*_(1,35)_ = 0.00, *p* > 0.05, *η* ^2^ = 0.000
	Post	158.75 (±36.67)	151.62 (±31.74)	
Grid Span (score)	Pre	5.63 (±0.72)	4.75 (±0.91)	T: *F*_(1,34)_ = 0.09, *p* > 0.05, *η* ^2^ = 0.003 G^∗^T: *F*_(1,34)_ = 1.86, *p* > 0.05, *η* ^2^ = 0.052
	Post	5.31 (±1.14)	4.95 (±1.00)	
Switching “single” (ms)	Pre	839.75 (±120.60)	808.43 (±84.38)	T: *F*_(1,35)_ = 0.82, *p* > 0.05, *η* ^2^ = 0.023 T^∗^G: *F*_(1,35)_ = 0.24, *p* > 0.05, *η* ^2^ = 0.007 Tr: *F*_(1.44,50.22)_ = 37.39, *p* < 0.00, *η* ^2^ = 0.517 Tr^∗^G: *F*_(1.44,50.22)_ = 0.02, *p* > 0.05, *η* ^2^ = 0.000 T^∗^Tr: *F*_(1.61,56.33)_ = 3.71, *p*=0.04, *η* ^2^ = 0.096 G^∗^T^∗^ Tr: *F*_(1.61,56.33)_ = 0.43, *p* > 0.05, *η* ^2^ = 0.012
	Post	790.35 (±105.56)	758.51 (±122.16)	
Switching “nonswitch” (ms)	Pre	884.69 (±157.93)	840.80 (±102.71)	
	Post	855.24 (±123.44)	836.59 (±156.47)	
Switching “switch” (ms)	Pre	951.24 (±146.64)	905.39 (±153.74)	
	Post	940.00 (±144.86)	932.36 (±174.00)	
TUG (s)	Pre	8.70 (±1.40)	8.52 (±1.67)	T: *F*_(1,35)_ = 0.57, *p* > 0.05, *η* ^2^ = 0.016 G^∗^T: *F*_(1,35)_ = 3.72, *p* > 0.05, *η* ^2^ = 0.096
	Post	8.37 (±1.41)	8.67 (±1.75)	

Data from the attention window test are presented in Figure 2.

## Supplementary Materials

Supplementary Materials Table S1: Mean values (±standard deviation), 95% confidence interval (CI), and statistics for intervention (int) and control (con) groups.
