# Supplementary material for: Can Driving-Simulator Training Enhance Visual Attention, Cognition, and Physical Functioning in Older Adults?
Source: J Aging Res. 2018 Feb 7;2018:7547631. doi: 10.1155/2018/7547631 (PMC5821998; doi:10.1155/2018/7547631)
Supplement: Supplementary Materials — Table S1: Mean values (±standard deviation), 95% confidence interval (CI), and statistics for intervention (int) and control (con) groups. [file 7547631.f1.docx]

*Table S1: Supplementary table including mean values (±standard deviation), 95%-confidence interval (CI), and statistics for intervention (int) and control (con) group.*

| Test | | int | 95%-CI_int_ | con | 95%-CI_con_ | Statistics |
| --- | --- | --- | --- | --- | --- | --- |
| Precue “false” [ms] | Pre | 371.63(±64.96) | 336.37-406.52 | 393.75(±71.48) | 363.29-424.21 | T: F_(1,35)_ = 0.15, p > 0.05, ɳ^2^ = 0.004;  T*G: F_(1,35)_ = 0.05, p > 0.05, ɳ^2^ = 0.001;  C: F_(1.686,59.012)_ = 19.75, p < 0.00, ɳ^2^ = 0.361;  C*G: F_(1.686,59.012)_ = 1.68, p > 0.05, ɳ^2^ = 0.046;  T*C: F_(1.703,59.618)_ = 0.46, p > 0.05, ɳ^2^ = 0.013;  G*T*C: F_(1.703,59.618)_ = 0.79, p > 0.05, ɳ^2^ = 0.022 |
|  | Post | 374.38(±76.03) | 335.31-413.44 | 391.77(±77.68) | 357.67-425.88 |  |
| Precue “neutral” [ms] | Pre | 362.88(±61.16) | 332.05-393.69 | 377.48(±60.41) | 350.57-404.38 |  |
|  | Post | 355.16(±50.90) | 324.69-385.62 | 372.71(±66.04) | 346.12-399.30 |  |
| Precue “correct” [ms] | Pre | 358.92(±68.31) | 331.17-386.68 | 357.26(±41.64) | 333.04-381.49 |  |
|  | Post | 348.70(±53.34) | 318.69-378.71 | 359.86(±63.12) | 333.67-386.05 |  |
| D2 [score] | Pre | 141.13(±37.81) | 122.24-160.01 | 134.29(±36.76) | 117.80-150.77 | T: F_(1,35)_ = 13.25, p < 0.01, ɳ^2^ = 0.275;  G*T: F_(1,35)_ = 0.00, p > 0.05, ɳ^2^ = 0.000 |
|  | Post | 158.75(±36.67) | 141.52-175.98 | 151.62(±31.74) | 136.58-166.66 |  |
| Grid Span [score] | Pre | 5.63(±0.72) | 5.20-6.05 | 4.75(±0.91) | 4.37-5.13 | T: F_(1,34)_ = 0.09, p > 0.05, ɳ^2^ = 0.003;  G*T: F_(1,34)_ = 1.86, p > 0.05, ɳ^2^ = 0.052 |
|  | Post | 5.31(±1.14) | 4.77-5.85 | 4.95(±1.00) | 4.47-5.43 |  |
| Switching “single” [ms] | Pre | 839.75(±120.60) | 788.24-891.26 | 808.43(±84.38) | 763.47-853.39 | T: F_(1,35)_ = 0.82, p > 0.05, ɳ^2^ = 0.023;  T*G: F_(1,35)_ = 0.24, p > 0.05, ɳ^2^ = 0.007;  Tr: F_(1.435,50.215)_ = 37.39, p < 0.00, ɳ^2^ = 0.517;  Tr*G: F_(1.435,50.215)_ = 0.02, p > 0.05, ɳ^2^ = 0.000;  T*Tr: F_(1.609,56.332)_ = 3.71, p = 0.04, ɳ^2^ = 0.096;  G*T* Tr: F_(1.609,56.332)_ = 0.43, p > 0.05, ɳ^2^ = 0.012 |
|  | Post | 790.35(±105.56) | 731.81-848.88 | 758.51(±122.16) | 707.41-809.60 |  |
| Switching “non-switch” [ms] | Pre | 884.69(±157.93) | 819.07-950.31 | 840.80(±102.71) | 783.52-898.08 |  |
|  | Post | 855.24(±123.44) | 782.54-927.94 | 836.59(±156.47) | 773.13-900.05 |  |
| Switching “switch” [ms] | Pre | 951.24(±146.64) | 874.73-1027.74 | 905.39(±153.74) | 838.62-972.17 |  |
|  | Post | 940.00(±144.86) | 857.70-1022.29 | 932.36(±174.00) | 860.53-1004.19 |  |
| TUG [s] | Pre | 8.70(±1.40) | 7.91-9.49 | 8.52(±1.67) | 7.82-9.21 | T: F_(1,35)_ = 0.57, p > 0.05, ɳ^2^ = 0.016;  G*T: F_(1,35)_ = 3.72, p > 0.05, ɳ^2^ = 0.096 |
|  | Post | 8.37(±1.41) | 7.55-9.18 | 8.67(±1.75) | 7.95-9.38 |  |
| AW (diagonal) [%] | Pre | 0.37(±0.09) | 0.32-0.43 | 0.30(±0.12) | 0.26-0.35 | T: F_(1,35)_ = 0.00, p > 0.05, ɳ^2^ = 0.000;  T*G: F_(1,35)_ = 0.14, p > 0.05, ɳ^2^ = 0.004 |
|  | Post | 0.38(±0.10) | 0.32-0.44 | 0.29(±0.12) | 0.25-0.35 |  |
| AW (horizontal) [%] | Pre | 0.36(±0.09) | 0.30-0.41 | 0.31(±0.11) | 0.26-0.35 | T: F_(1,35)_ = 0.65, p > 0.05, ɳ^2^ = 0.018;  T*G: F_(1,35)_ = 4.46, p = 0.04, ɳ^2^ = 0.113 |
|  | Post | 0.39(±0.09) | 0.34-0.45 | 0.29(±0.12) | 0.24-0.34 |  |
| AW (vertical) [%] | Pre | 0.37(±0.05) | 0.31-0.42 | 0.31(±0.12) | 0.26-0.35 | T: F_(1,35)_ = 0.01, p > 0.05, ɳ^2^ = 0.000;  T*G: F_(1,35)_ = 0.01, p > 0.05, ɳ^2^ = 0.000 |
|  | Post | 0.37(±0.09) | 0.31-0.42 | 0.31(±0.12) | 0.26-0.36 |  |
